# The Association Between Quantitative Flow Ratio and Intravascular Imaging-defined Vulnerable Plaque Characteristics in Patients With Stable Angina and Non-ST-segment Elevation Acute Coronary Syndrome

**DOI:** 10.3389/fcvm.2021.690262

**Published:** 2021-06-30

**Authors:** Wenjie Zuo, Renhua Sun, Xiaoguo Zhang, Yangyang Qu, Zhenjun Ji, Yamin Su, Rui Zhang, Genshan Ma

**Affiliations:** ^1^Department of Cardiology, Zhongda Hospital, School of Medicine, Southeast University, Nanjing, China; ^2^Department of Cardiology, The First People's Hospital of Yancheng, Yancheng, China

**Keywords:** quantitative flow ratio (QFR), optical coherence tomography (OCT), intravascular ultrasound (IVUS), fractional flow reserve (FFR), plaque vulnerability

## Abstract

**Background:** This study aimed to examine whether quantitative flow ratio (QFR), an angiography-based computation of fractional flow reserve, was associated with intravascular imaging-defined vulnerable plaque features, such as thin cap fibroatheroma (TCFA) in patients with stable angina, and non-ST-segment elevation acute coronary syndrome.

**Methods:** Patients undergoing optical coherence tomography (OCT) or intravascular ultrasound (IVUS) examinations were identified from two prospective studies and their interrogated vessels were assessed with QFR. Lesions in the OCT cohort were classified into tertiles: QFR-T1 (QFR ≤ 0.85), QFR-T2 (0.85 < QFR ≤ 0.93), and QFR-T3 (QFR > 0.93). Lesions in the IVUS cohort were classified dichotomously as low or high QFR groups.

**Results:** This *post-hoc* analysis included 132 lesions (83 for OCT and 49 for IVUS) from 126 patients. The prevalence of OCT-TCFA was significantly higher in QFR-T1 (50%) than in QFR-T2 (14%) and QFR-T3 (19%) (*p* = 0.003 and 0.018, respectively). Overall significant differences were also observed among tertiles in maximum lipid arc, thinnest fibrous cap thickness, and minimal lumen area (*p* = 0.017, 0.040, and <0.001, respectively). Thrombus was more prevalent in QFR-T1 (39%) than in QFR-T2 (3%), and QFR-T3 (12%) (*p* = 0.001 and 0.020, respectively). In the multivariable analysis, QFR ≤ 0.80 remained as a significant determinant of OCT-TCFA regardless of the presence of NSTE-ACS and the level of low-density lipoprotein cholesterol (adjusted OR: 4.387, 95% CI 1.297–14.839, *p* = 0.017). The diagnostic accuracy of QFR was moderate in identifying lesions with OCT-TCFA (area under the curve: 0.72, 95% CI 0.58–0.86, *p* = 0.003). In the IVUS cohort, significant differences were found between two groups in minimal lumen area and plaque burden but not in the distribution of virtual histology (VH)-TCFA (*p* = 0.025, 0.036, and 1.000, respectively).

**Conclusions:** Lower QFR was related to OCT-defined plaque vulnerability in angiographically mild-to-intermediate lesions. The QFR might be a useful tool for ruling out high-risk plaques without using any pressure wire or vasodilator.

## Introduction

The fractional flow reserve (FFR) is now widely accepted as an essential tool in assessing the physiological severity of coronary stenosis, and guiding decision-making for myocardial revascularization (1, 2). The numeric value of FFR was demonstrated to have a continuous and independent relationship with future adverse cardiac events thus it may partially reflect the extent of plaque vulnerability (i.e., tendency to rupture) (3). However, the invasive FFR measurement is usually associated with a prolonged procedure time and increased medical expenses (e.g., costs of pressure wires and hyperemic agents) (4). For this reason, angiography-based quantitative flow ratio (QFR) has been recently proposed and can be used as a surrogate indicator of functional ischemia because of its strong agreement with FFR (5–7).

Postmortem studies found that a plaque prone to rupture is typically characterized by a large lipid or necrotic core that is covered by a thin fibrous cap and, introduced the concept of thin cap fibroatheroma (TCFA) to describe this atherosclerotic plaque type (8, 9). This atherosclerotic plaque type, termed as TCFA, is the most commonly used to describe the plaque vulnerability. Other vulnerable indicators include minimal lumen area, plaque burden, macrophage infiltration, and lipid arc circumferential extension (10, 11). These vulnerable characteristics can be visualized *in vivo* by intravascular imaging modalities, such as virtual histology intravascular ultrasound (VH-IVUS) and high-resolution optical coherence tomography (OCT) (12). Emerging evidence has established the association between high-risk plaque features and the presence of adverse coronary events (11, 13). Recently, QFR was recognized to be associated with the presence of OCT-TCFA in stable patients, and non-culprit lesions in patients with acute coronary syndrome (ACS) (14). However, the majority of the included lesions (89%) were from patients with stable coronary artery disease and this association needs to be verified in a broader spectrum of patients. Additionally, discrepant results were reported in previous studies examining whether plaque characteristics assessed by OCT (14–16) or VH-IVUS (17–20) had an impact on coronary hemodynamics. Hence, the association between coronary physiology and OCT/IVUS-defined plaque vulnerability remains elusive and warrants more evidence. Given this background, we sought to further investigate the relationships between QFR and lesion-specific morphological characteristics detected by OCT or IVUS, not only in patients presenting stable angina but also in culprit lesions from patients with medically stabilized non-ST-segment elevation ACS (NSTE-ACS).

## Materials and Methods

### Study Design and Population

This *post-hoc* analysis screened 164 participants between 2018 and 2020 from two prospective studies at Zhongda Hospital, School of Medicine, Southeast University, Nanjing, China. One of the studies was a single-center, randomized, controlled trial that assigned patients with angiographically mild-to-intermediate coronary lesions (30–70% diameter stenosis by visual estimation) to FFR-guided, IVUS-guided, or OCT-guided revascularization. The other one was an observational study designed to explore potential biomarkers related to OCT-defined plaque vulnerability. All patients who underwent intravascular imaging (OCT/IVUS) in the two studies were eligible for subsequent QFR computation. Exclusion criteria included ST-segment elevation myocardial infarction (STEMI), myocardial bridge, poor image quality, and insufficient angiographic projections for QFR assessment. The two studies were performed following the Declaration of Helsinki and their protocols were approved by the local institutional ethics committee. All participants provided written informed consent at the time of enrollment.

### Coronary Catheterization

Each patient received standard coronary angiography *via* the radial route with a 5- or 6-French diagnostic catheter after an intracoronary infusion of nitroglycerin (0.1–0.2 mg). Angiographic images were routinely recorded at 15 frames/s using the radiographic imaging system (AXIOM Artis, Siemens, Erlangen, Germany), and several projection views were attempted to avoid severe overlapping or excessive foreshortening. The OCT/IVUS imaging was conducted before revascularization for interrogated vessels or during the diagnostic process in patients who were deferred for stenting.

### OCT Image Acquisition and Analysis

All OCT images were acquired using a frequency-domain C7-XR^™^ OCT system (St. Jude Medical, Westford, MA, USA) according to previous protocols (21). In brief, the OCT imaging catheter (C7 Dragonfly, St. Jude Medical, Westford, MA, USA) was advanced distal to the target lesion, and then iso-osmotic contrast media was continuously infused into the artery to achieve blood clearance when the catheter was pulled back automatically at a steady rate of 25 mm/s (100 frames/s). All OCT images were digitally documented in our database and analyzed offline with a dedicated workstation (1.0 mm interval) by experienced readers who were unaware of clinical data.

**Figure 1:**
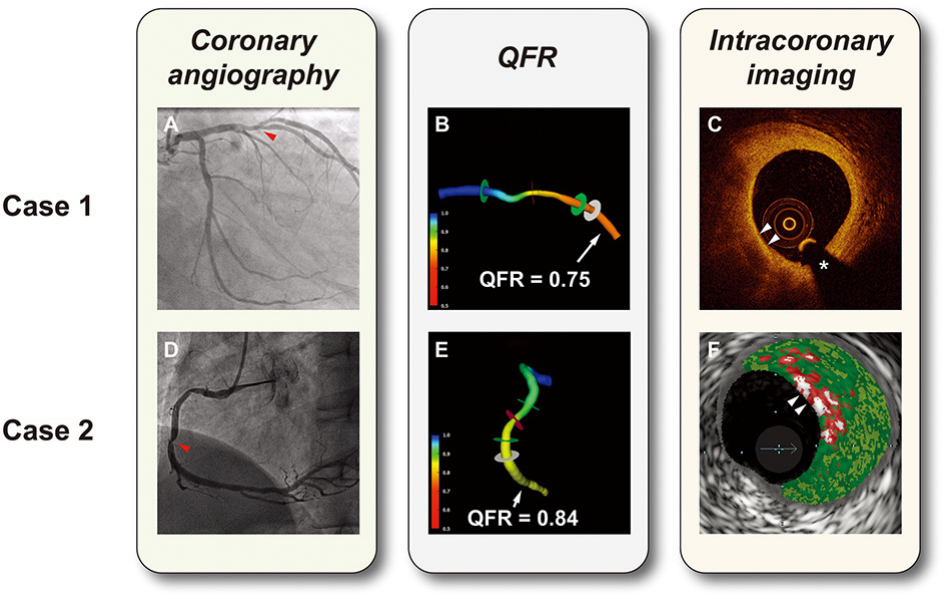
Representative cases of hemodynamic and intravascular evaluations. **(A)** Coronary angiography showed a lesion with 50% diameter stenosis in the left anterior descending artery (the red arrow). **(B)** 3D vessel reconstruction with a QFR of 0.75. **(C)** OCT-TCFA: a lipid-rich plaque with the thinnest fibrous cap <65 *μ*m (white arrows). **(D)** Coronary angiography showed a lesion with 50% diameter stenosis in the right coronary artery (the red arrow). **(E)** 3D vessel reconstruction with a QFR of 0.84. **(F)** VH-caTCFA (white arrows): necrotic core is coded as red, dense calcium as white, fibrous tissue as dark green, and fibrofatty tissue as light green. ^*^guidewire artifact. QFR, quantitative flow ratio; OCT-TCFA, optical coherence tomography-thin cap fibroatheroma; VH-caTCFA, virtual histology-calcified thin cap fibroatheroma.

Interrogated vessels were assessed both qualitatively and quantitatively according to consensus standards for OCT analysis (22). Plaques were categorized into fibrous (high backscattering with a relatively homogenous signal) or lipid (a signal-poor region with poorly defined or diffuse borders) (23, 24). Lipid length was measured longitudinally and the lipid arc was also measured through the entire length of plaques. A plaque was considered to be lipid-rich when its lipid arc was >90° in any cross-sectional image. Lipid index was calculated as the mean lipid arc × lipid length (25). The fibrous cap was often presented as a signal-rich, tissue cap overlying a signal-poor region in OCT images. The OCT-TCFA was characterized as a lipid-rich plaque with its minimal fibrous cap thickness of ≤65 *μ*m (Figure 1) (8, 25). Macrophage infiltration was identified as signal-rich, distinct, or confluent punctate regions with a higher intensity than background speckle noise (22, 26). Microchannels were defined as signal-poor, sharply delineated voids that appeared in multiple consecutive frames (22, 27). Cholesterol crystals were seen by OCT as thin, linear, and high-intensity regions within the plaque (22, 28). Plaque rupture was defined by the presence of fibrous cap disruption and cavity formation (29). Plaque erosion was defined by the presence of thrombus on an irregular luminal surface without clear evidence of cap rupture (30).

### IVUS Image Acquisition and Analysis

IVUS imaging was performed by positioning a 20-MHz Eagle Eye Gold^™^ IVUS catheter (Volcano Corp., Rancho Cordova, CA, USA) distal to the lesion and using a motorized pullback at 0.5 mm/s. All IVUS images were recorded on discs and subsequently analyzed with the offline S5^™^ workstation by independent technicians who were blinded to the clinical characteristics of patients. Grayscale IVUS measurement and VH-IVUS tissue characterization were performed according to validated criteria for IVUS analysis (31, 32). Cross-sectional areas (CSA) of the external elastic membrane (EEM) at the narrowest site and minimal lumen area (MLA) were measured by a semi-automatic algorithm, and the contour was corrected manually if necessary. Plaque burden was calculated as: 100% × (EEM CSA—MLA)/EEM CSA. Remodeling index were calculated as: EEM CSA at the MLA site/EEM at the reference site. The EEM CSA at the reference site was estimated as the average value of proximal and distal reference EEM CSAs within 5 mm to the lesion (20). Positive or negative remodeling was identified when the remodeling index >1.05 or <0.95, respectively (33).

The four main plaque components were marked in color and their relative percentages at the MLA site were measured by VH-IVUS: fibrous (green), fibro-fatty (yellow), dense calcium (white), and necrotic core (red) (32). The virtual histology-TCFA (VH-TCFA) was defined as a lesion with both >10% confluent necrotic core in contact with lumen and plaque burden >40% for three consecutive frames (10, 13, 32). In the analysis of OCT and IVUS images, any disagreement between readers was resolved by consensus, and when necessary, was determined by a third investigator.

### 3D-QCA and QFR Computation

The 3-dimensional quantitative coronary angiography (3D-QCA) and subsequent QFR computation were performed offline by two certified technicians who used the QFR workstation (AngioPlus, Pulse Medical Imaging Technology, Shanghai Co., Ltd., Shanghai, China) as previously described (6, 34), and their discordance was resolved in a similar way to OCT/IVUS analysis. Two end-diastole angiographic frames from different angles ≥25° were selected for the 3D reconstruction of the interrogated vessel. Arterial contours were detected automatically by algorithms and manual correction was allowed when image quality was sub-optimal. The reference vessels were defined as visually normal segments proximal or distal to the lesion of interest. Minimal lumen diameter, percent diameter stenosis, and lesion length were measured using the 3D-QCA module. The measurement of contrast flow velocity was undertaken using a frame count method integrated into the software and contrast-flow QFR value was then generated (5). All 3D-QCA and QFR analyses were based on previously documented angiograms and technicians were blinded to patients' characteristics and OCT/IVUS parameters.

### Statistical Analysis

Histograms and Q-Q plots were used to examine whether continuous variables were consistent with a normal distribution. Continuous variables were expressed as mean ± standard deviation (SD) or median (interquartile range) as appropriate and were compared using the Student's *t*-test and Mann-Whitney *U* test, respectively. The differences among the tertiles were analyzed using the Kruskal-Wallis test followed by pairwise comparisons. Categorical variables were expressed as counts (percentages) and compared using the Chi-square test or Fisher's exact test as appropriate. Relationships between QFR and morphological parameters were assessed by Spearman's correlation analysis. Multivariable logistic regression analysis was used to exclude other potential confounding factors. Moreover, receiver-operating characteristic curve analyses were performed to determine the predictive ability of QFR for OCT-TCFA, VH-TCFA, OCT-MLA < 3.5 mm^2^, IVUS-MLA < 4 mm^2^, and plaque burden ≥70% (10, 11). The Youden index was used to identify the optimal cutoff values for QFR. All statistical analysis was performed using SPSS version 25.0 (IBM Corp., Armonk, NY, USA) and GraphPad Prism version 8.2.1 for macOS (GraphPad Software, San Diego, CA, USA). A two-tailed *P*-value < 0.05 indicated statistical significance.

## Results

### Baseline Characteristics of Included Patients and Lesions

**Figure 2:**
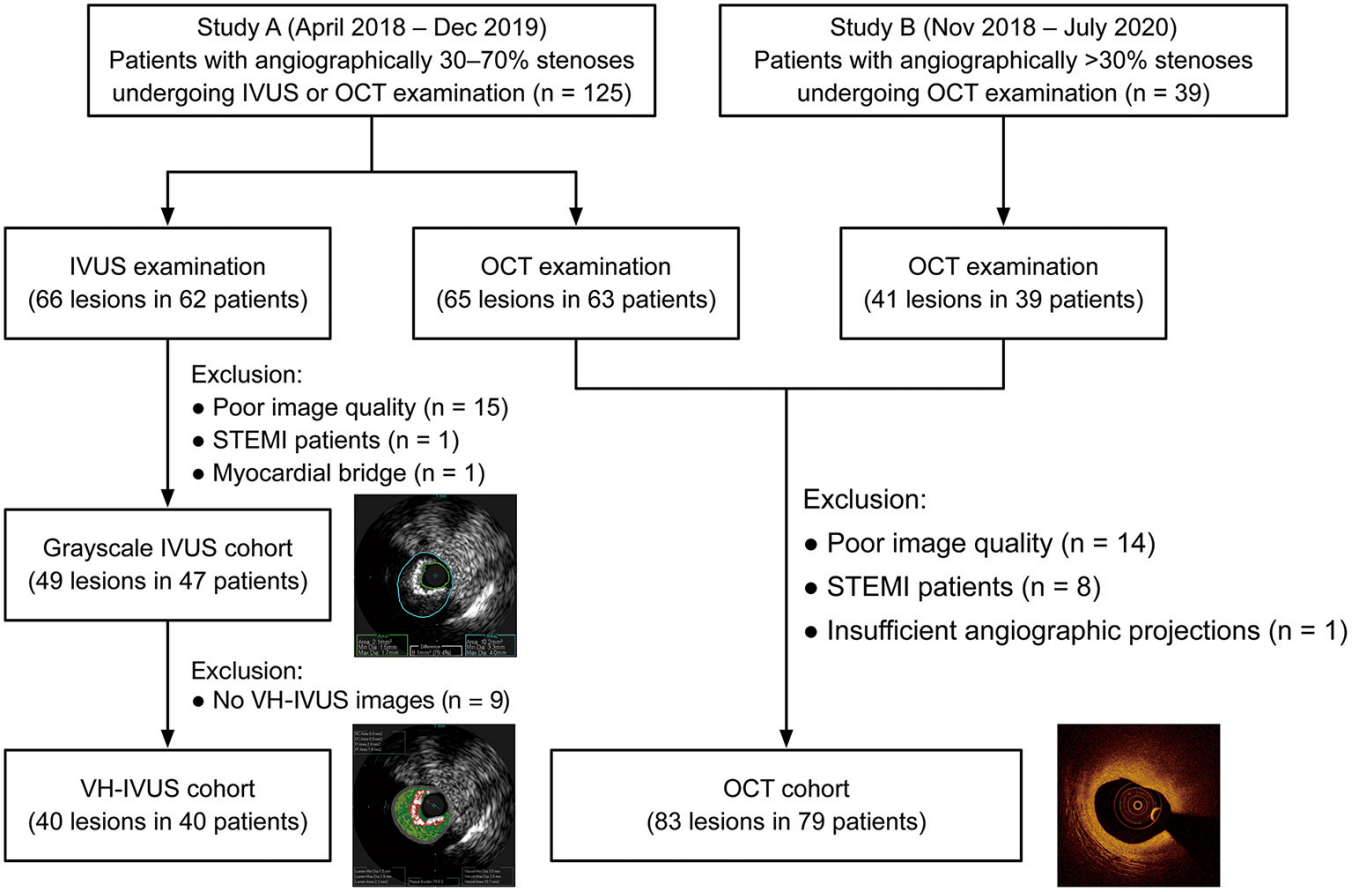
Study flow diagram. IVUS, intravascular ultrasound; OCT, optical coherence tomography; STEMI, ST-segment elevation myocardial infarction; VH-IVUS, virtual histology intravascular ultrasound.

In total, 132 lesions (83 for OCT and 49 for IVUS) from 126 patients were included for the final analysis (Figure 2). The baseline characteristics of included patients and lesions are summarized in Table 1. For the overall population, there was a relatively high proportion of patients with NSTE-ACS (63%) but with a preserved left ventricular ejection fraction [median, 69% (66–74%)]. The median % diameter stenosis and median QFR value were 42% (36–49%) and 0.88 (0.83–0.95), respectively. According to the QFR value, OCT-assessed lesions were divided into tertiles as follows: lowest tertile (QFR-T1; QFR ≤ 0.85; *n* = 28), middle tertile (QFR-T2; 0.85 < QFR ≤ 0.93; *n* = 29), and highest tertile (QFR-T3; QFR > 0.93; *n* = 26). The IVUS-assessed lesions were divided into low QFR (QFR ≤ 0.87; *n* = 26), and high QFR (QFR > 0.87; *n* = 23) groups. Of 49 IVUS-assessed lesions, 40 (82%) were eligible for subsequent tissue characterization and were regarded as a VH-IVUS subgroup (Supplementary Table 1). For OCT and IVUS cohorts, 23 and two lesions were suspected to be in the infarct-related arteries, respectively.

**Table 1:** Baseline characteristics of included patients and lesions.

	**OCT cohort**	**IVUS cohort**
**Clinical and demographic characteristics**		
Patient No.	79	47
Age, years	61.5 ± 9.7	63.5 ± 10.0
Male	46 (58.2)	29 (61.7)
Body mass index, kg/m^2^	25.2 ± 3.6	25.1 ± 4.6
Hypertension	57 (72.2)	34 (72.3)
Diabetes mellitus	17 (21.5)	12 (25.5)
Dyslipidemia	16 (20.3)	13 (27.7)
Smoking	18 (22.8)	11 (23.4)
Previous MI	1 (1.3)	1 (2.1)
Previous PCI	7 (8.9)	3 (6.4)
LVEF, %	69.5 (66.0–74.0)	69.1 (66.0–75.0)
Triglyceride, mmol/L	1.4 (0.9–2.2)	1.3 (1.0–2.1)
Total cholesterol, mmol/L	4.2 (3.7–5.0)	4.3 (3.4–4.9)
LDL cholesterol, mmol/L	2.4 (1.9–3.1)	2.4 (2.0–3.1)
HDL cholesterol, mmol/L	1.2 (1.0–1.4)	1.2 (1.0–1.4)
**Symptoms**		
Stable angina	26 (32.9)	20 (42.6)
Unstable angina	29 (36.7)	25 (53.2)
NSTEMI	24 (30.4)	2 (4.2)
**Medications**		
Aspirin	23 (29.1)	12 (25.5)
Statins	20 (25.3)	11 (23.4)
*β*-blockers	12 (15.2)	5 (10.6)
ACEIs/ARBs	19 (24.1)	15 (31.9)
Calcium channel blockers	33 (41.8)	15 (31.9)
**Interrogated vessel characteristics**		
Lesion No.	83	49
**Lesion location**		
LAD	62 (74.7)	39 (79.6)
LCX	10 (12.0)	3 (6.1)
RCA	11 (13.3)	7 (14.3)
**3D-QCA**		
Diameter stenosis, %	40.4 (34.3–49.4)	45.6 (37.5–48.4)
Lesion length, mm	15.9 (11.4–22.6)	20.3 (12.0–26.4)
MLD, mm	1.6 (1.3–1.9)	1.6 (1.5–1.9)
QFR	0.88 (0.83–0.95)	0.87 (0.82–0.93)

*Values are expressed as n (%) or mean ± standard deviation or median (interquartile range). ACEI, angiotensin-converting enzyme inhibitor; ARB, angiotensin receptor inhibitor; HDL, high-density lipoprotein; IVUS, intravascular ultrasound; LAD, left anterior descending artery; LCX, left circumflex artery; LDL, low-density lipoprotein; LVEF, left ventricular ejection fraction; MI, myocardial infarction; MLD, minimal lumen diameter; NSTEMI, non-ST-segment elevation myocardial infarction; OCT, optical coherence tomography; PCI, percutaneous coronary intervention; QCA, quantitative coronary angiography; QFR, quantitative flow ratio; RCA, right coronary artery*.

### Association Between OCT Findings and QFR

**Figure 3:**
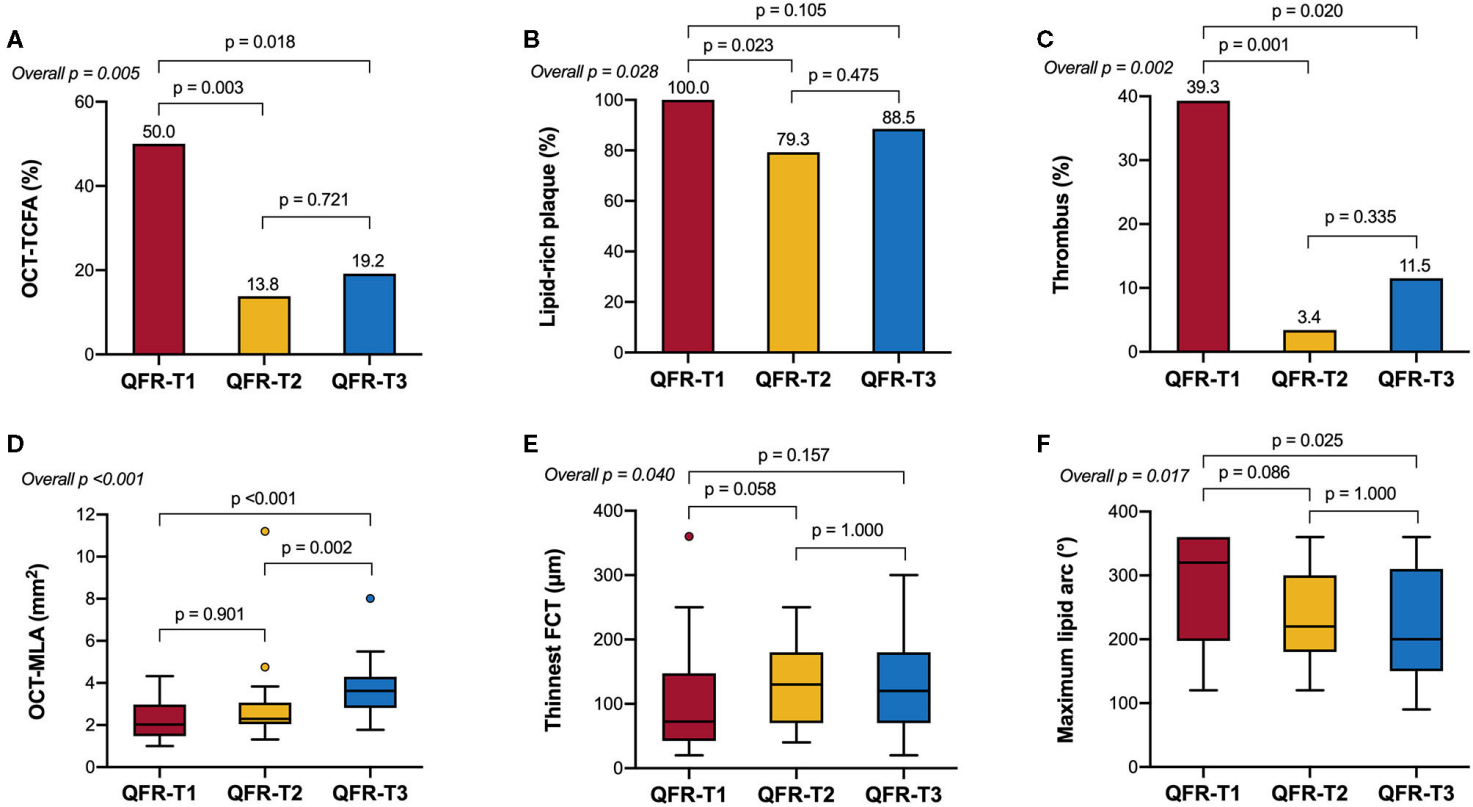
OCT-derived morphological characteristics stratified by QFR tertiles. **(A)** The prevalence of OCT-TCFA was the highest in QFR-T1; however, it was not significantly different between QFR-T2 and QFR-T3. **(B)** The prevalence of lipid-rich plaques was the highest in QFR-T1, although no significance was observed in pairwise comparisons after Bonferroni adjustment. **(C)** Compared with the other tertiles, thrombosis is more likely to occur in QFR-T1. **(D)** QFR-T3 had a significantly larger OCT-MLA than the other tertiles. **(E)** There was an overall difference in the thinnest FCT among tertiles but no pairwise comparison was significant. **(F)** The maximum lipid arc was significantly different among tertiles. FCT, fibrous cap thickness; OCT-MLA, optical coherence tomography-minimal lumen area; other abbreviations as in Figure 1.

Table 2 and Figure 3 show the comparison of OCT-defined lesion morphology among QFR tertiles. Among the three groups, OCT-derived MLA and % area stenosis showed a significant, graded change (*p* < 0.001 for both). The prevalence of lipid-rich plaques in the lowest QFR tertile was significantly higher than that in the other two tertiles (*p* = 0.028). The maximum lipid arc was 320° (198–360°) in the QFR-T1, significantly higher than the other tertiles (*p* = 0.017). The QFR-T1 was associated with a higher frequency of TCFAs (*p* = 0.005), and a thinner fibrous cap thickness (*p* = 0.04) as compared to the other tertiles. Moreover, thrombus formation is more likely to occur in the QFR-T1 than the other tertiles (*p* = 0.002). Lesion characteristics according to the presence or absence of OCT-TCFA was shown in Supplementary Table 2.

**Table 2:** OCT characteristics according to QFR tertiles.

	**Lowest tertile** **(T1, *n* = 28)**	**Middle tertile** **(T2, *n* = 29)**	**Highest tertile** **(T3, *n* = 26)**	***p*****-value**			
				**Overall**	**T1 vs. T2**	**T1 vs. T3**	**T2 vs. T3**
MLA, mm^2^	2.03 (1.47–2.97)	2.30 (2.05–3.07)	3.63 (2.82–4.30)	<0.001	0.901	<0.001	0.002
Area stenosis, %	68.1 ± 12.8	60.1 ± 9.0	54.2 ± 13.7	<0.001	0.033	<0.001	0.170
Lipid-rich plaques	28 (100.0)	23 (79.3)	23 (88.5)	0.028	0.023	0.105	0.475
Average lipid arc, °	155 (135–202)	153 (107–185)	126 (105–165)	0.019	0.606	0.015	0.433
Maximum lipid arc, °	320 (198–360)	220 (180–300)	200 (150–310)	0.017	0.086	0.025	1.000
Lipid length, mm	18.5 (11.6–22.4)	12.5 (8.0–21.0)	12.0 (8.0–18.0)	0.223	–	–	–
Lipid index, ° × mm	2,527 (1,973–3,844)	1,756 (1,085–3,648)	1,574 (578–2,415)	0.058	–	–	–
Thinnest FCT, μm	73 (43–148)	150 (70–190)	120 (70–180)	0.040	0.058	0.157	1.000
TCFAs	14 (50.0)	4 (13.8)	5 (19.2)	0.005	0.003	0.018	0.721
Plaque rupture	4 (14.3)	0 (0.0)	1 (3.8)	0.057	0.052	0.353	0.473
Plaque erosion	6 (21.4)	1 (3.4)	3 (11.5)	0.101	0.052	0.470	0.335
Thrombus	11 (39.3)	1 (3.4)	3 (11.5)	0.002	0.001	0.020	0.335
Calcification	14 (50.0)	14 (48.3)	9 (34.6)	0.463	0.896	0.253	0.305
Calcified nodules	2 (7.1)	1 (3.4)	2 (7.7)	0.735	0.611	1.000	0.598
Microchannels	9 (32.1)	9 (31.0)	11 (42.3)	0.634	0.928	0.440	0.386
Macrophage accumulation	15 (53.6)	9 (31.0)	10 (38.5)	0.213	0.085	0.266	0.563
Cholesterol crystals	10 (35.7)	11 (37.9)	8 (30.8)	0.852	0.862	0.700	0.577

*Values are expressed as n (%) or mean ± standard deviation or median (interquartile range). FCT, fibrous cap thickness; MLA, minimal lumen area; TCFA, thin cap fibroatheroma; other abbreviations as Table 1*.

In Spearman's correlation analyses (Supplementary Figure 1), QFR was significantly related to OCT-derived MLA and % area stenosis (*ρ* = 0.537 and −0.512, respectively; *p* < 0.001 for both). There was an inverse correlation between QFR and parameters of lipid-rich plaques, including maximum lipid arc (*ρ* = −0.360, *p* = 0.002), lipid length (*ρ* = −0.242, *p* = 0.038), and lipid index (*ρ* = −0.333, *p* = 0.004). Additionally, QFR was moderately correlated to the thinnest fibrous cap thickness (*ρ* = 0.315, *p* = 0.006). In the multivariable analysis, QFR ≤ 0.80 remained as a significant determinant of OCT-TCFA regardless of the presence of NSTE-ACS and the level of low-density lipoprotein cholesterol (adjusted odds ratio = 4.387, 95% CI: 1.297–14.839, *p* = 0.017).

### Association Between IVUS Findings and QFR

**Figure 4:**
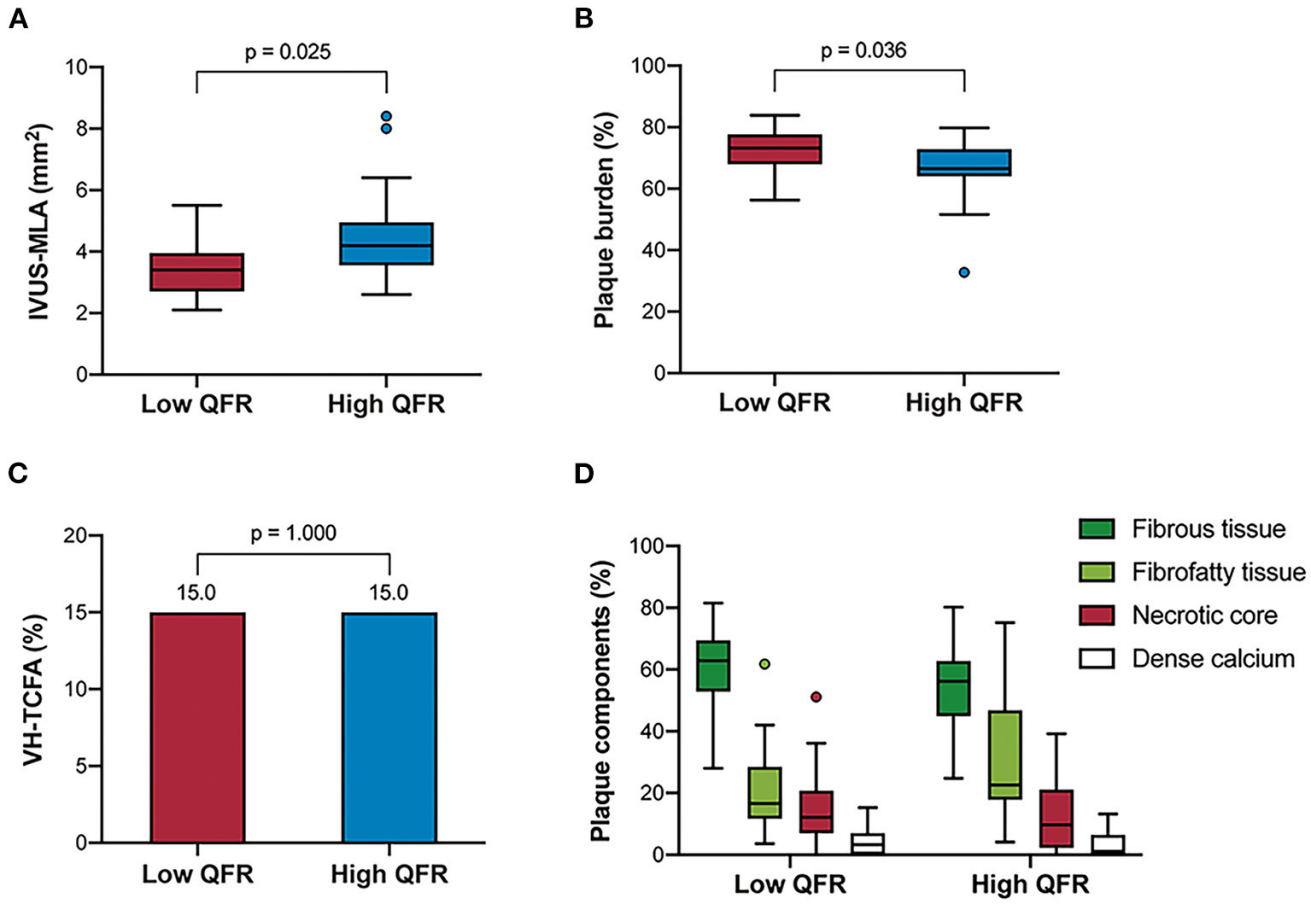
IVUS-derived morphological characteristics stratified by QFR groups. The grayscale IVUS cohort (*n* = 49) showed that the high QFR group had a larger IVUS-MLA **(A)** but a smaller plaque burden **(B)** compared to the low QFR group. However, the VH-IVUS subgroup (*n* = 40) showed that there was no significant difference in the frequency of VH-TCFA, **(C)** and plaque components **(D)** between the groups. VH-TCFA, virtual histology-thin cap fibroatheroma; other abbreviations as in Figures 1, 2.

Table 3 and Figure 4 show the comparison of IVUS-defined plaque morphology between the two groups divided by QFR values. The low QFR group was associated with an increased plaque burden [73.2% (68.0–77.7 %) vs. 66.5 % (64.0–72.9 %), *p* = 0.036], and a smaller IVUS-derived MLA [3.5 mm^2^ (2.8–4.1 mm^2^) vs. 4.1 (3.4–4.9 mm^2^)] compared to its counterparts. The Spearman's correlation analysis also confirmed the significant association between QFR and the two morphological parameters (*ρ* = 0.426, *p* = 0.002 for IVUS-derived MLA; *ρ* = −0.413, *p* = 0.003 for plaque burden) (Supplementary Figure 2). In the VH-IVUS subgroup, lesions were divided into the low QFR group (QFR ≤ 0.88; *n* = 20), and high QFR group (QFR > 0.88; *n* = 20). However, no significant difference was found in the absolute values or relative percentages of the four main plaque components. Similarly, the distribution of VH-TCFA was not statistically different between the two groups (15 vs. 15%, *p* = 1.000). Lesion characteristics according to the presence or absence of VH-TCFA was shown in Supplementary Table 3.

**Table 3:** IVUS characteristics according to QFR groups.

	**Low QFR**	**High QFR**	***p*-value**
**Grayscale IVUS lesions (*****n*** **=** **49)**			
Lesion No.	26	23	–
Grayscale IVUS findings			
EEM CSA, mm^2^	12.3 (10.6–14.7)	12.4 (10.8–16.1)	0.515
Plaque + media, mm^2^	8.9 (7.5–10.7)	8.7 (7.0–10.3)	0.771
Plaque burden, %	73.2 (68.0–77.7)	66.5 (64.0–72.9)	0.036
MLA, mm^2^	3.5 (2.8–4.1)	4.1 (3.4–4.9)	0.025
Reference EEM CSA, mm^2^	13.1 (11.2–14.5)	13.0 (10.3–17.0)	0.528
Remodeling index	0.96 (0.80–1.10)	0.92 (0.81–1.08)	0.787
Positive remodeling	11 (42.3)	7 (30.4)	0.390
Negative remodeling	13 (50.0)	13 (56.5)	0.648
**VH-IVUS lesions (*****n*** **=** **40)**			
Lesion No.	20	20	–
VH-IVUS findings			
Fibrous tissue, mm^2^	2.7 (2.0–4.5)	2.7 (1.8–4.0)	0.678
Fibrous tissue, %	62.9 (52.9–69.5)	56.2 (45.0–62.8)	0.134
Fibrofatty tissue, mm^2^	1.0 (0.6–1.8)	1.8 (0.6–2.5)	0.121
Fibrofatty tissue, %	16.6 (11.7–28.5)	22.7 (17.9–46.8)	0.142
Necrotic core, mm^2^	0.7 (0.3–1.2)	0.4 (0.2–1.2)	0.478
Necrotic core, %	12.2 (7.0–20.8)	9.7 (2.3–21.1)	0.398
Dense calcium, mm^2^	0.2 (0.0–0.4)	0.1 (0.0–0.2)	0.398
Dense calcium, %	3.3 (0.1–7.1)	1.1 (0.0–6.5)	0.565
VH-TCFA	3 (15.0)	3 (15.0)	1.000

*Values are expressed as n (%) or median (interquartile range). CSA, cross-sectional area; EEM, external elastic membrane; VH-IVUS, virtual histology intravascular ultrasound; other abbreviations as Tables 1, 2*.

### Predictive Value of QFR for Plaque Morphology

**Figure 5:**
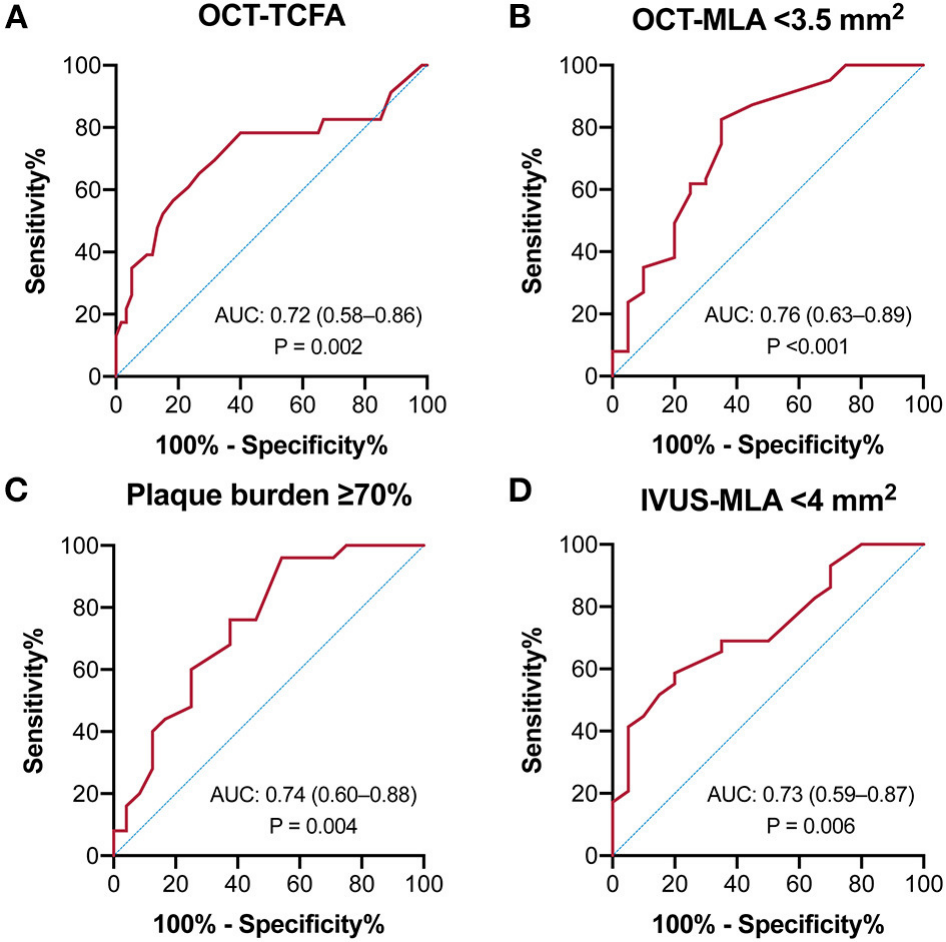
Receiver-operating characteristics curves of QFR to predict intravascular imaging-derived characteristics. The predictive ability of QFR was modest for **(A)** OCT-TCFA, **(B)** OCT-MLA < 3.5 mm^2^, **(C)** Plaque burden ≥70%, and **(D)** IVUS-MLA < 4 mm^2^.

As shown in Figure 5, the receiver operating curve analysis suggested that QFR had modest ability for recognition of an OCT-TCFA (AUC = 0.72, 95% CI: 0.58–0.86, *p* = 0.002) and a QFR value of ≤0.86 was the best cutoff point with a sensitivity of 65.2% (95% CI: 44.9–81.2%) and specificity of 73.3% (61.0–82.9%). Similarly, QFR showed good discrimination for the presence of OCT-MLA < 3.5 mm^2^, IVUS-MLA < 4 mm^2^, and plaque burden ≥70%, and their AUCs were 0.76 (95% CI: 0.63–0.89, *p* < 0.001), 0.73 (0.59–0.87, *p* = 0.006), and 0.74 (95% CI: 0.60–0.88, *p* = 0.004), respectively. Conversely, QFR had no significant discriminative ability for VH-TCFA (AUC = 0.54, 95% CI: 0.32–0.76, *p* = 0.733). The diagnostic performance of QFR for the evaluation of plaque morphology were detailed in Supplementary Table 4.

## Discussion

The present study performed a combined anatomical and physiological evaluation of angiographically mild-to-intermediate coronary lesions using the wire-free QFR, and intravascular imaging modalities (OCT or IVUS). In patients presenting stable angina and medically stabilized NSTE-ACS, QFR demonstrated significant correlations with several OCT or IVUS-derived morphological parameters and good diagnostic accuracy to detect TCFAs confirmed by OCT. In the OCT cohort, a lower QFR was associated with a smaller OCT-MLA, more severe area stenosis, a larger lipid arc, a thinner fibrous cap, and a higher prevalence of lipid-rich lesions and TCFAs. In the IVUS cohort, a lower QFR was associated with a smaller IVUS-MLA and a greater plaque burden; however, no association was found between QFR and VH-TCFA.

### QFR and OCT-Defined Plaque Vulnerability

A recent study indicated the link between QFR and the presence of OCT-TCFA in 327 *de novo* intermediate-to-severe coronary lesions but the majority of these lesions were from stable patients (14). Here, we further confirmed this relationship in an NSTE-ACS-dominated population and suggested the applicability of QFR in these patients. Notably, 23 lesions (27.7%) from the OCT cohort were suspected to be in infarct-related arteries. The reliability of hyperemic physiology in the setting of myocardial infarction has been questioned because of severe microvascular dysfunction, particularly in the culprit vessel of a patient with STEMI. However, patients with recent non-ST-segment elevation myocardial infarction (NSTEMI) have a different natural history from STEMI, and often present without coronary occlusion. In these medically stabilized patients, the vasodilator capacity may be preserved and FFR may not be influenced due to recovered microcirculation (35). Furthermore, Layland et al. (36) demonstrated that FFR measurement was accurate and reliable to diagnose reversible ischemia in both culprit and non-culprit lesions of patients with recent NSTEMI when compared with the stress cardiac magnetic resonance perfusion. Despite this fact, we performed a multivariable analysis to exclude the potential confounding effects of NSTE-ACS and recognized an independent relationship between QFR and OCT-TCFA. Unlike FFR, QFR is a non-hyperemic index and may be not influenced by microvascular obstruction. Subsequent studies would be necessary to investigate whether QFR-guided compared with angiography-guided revascularization in patients with ACS offers improved outcomes.

### QFR and IVUS-Defined Plaque Vulnerability

Several studies have examined the relationship between VH-IVUS-defined plaque features and physiological severity in coronary stenosis; however, their results are inconsistent. Most of the studies suggested that FFR correlated with IVUS-MLA and plaque burden but not with plaque compositions and TCFA (17–19). Conversely, a recent study by Sezer et al. (20) showed that necrotic volume, TCFA, and positive remodeling may have an impact on the hemodynamic outcome of intermediate lesions. The reason for such an inconsistency is likely to be the difference in the angiographic severity of the included lesions. Interestingly, we identified the irrelevance between VH-IVUS-defined plaque vulnerability and angiography-derived QFR in a spectrum of mild-to-intermediate lesions. This negative result likely arises from several factors. Compared with the OCT cohort, the VH-IVUS cohort had a smaller sample size that may be underpowered to detect a statistical difference and susceptible to confounding bias. In addition, the effect of morphological features on flow resistance might be impaired in angiographic mild stenosis (20). Plaque burden and IVUS-MLA seemed to have a much stronger influence on QFR than plaque morphology as indicated by our results. Besides, VH-IVUS may be suboptimal to identify a thin fibrous cap and surface irregularity due to its lower resolution than OCT (12). A head-to-head comparison indicated that the diagnostic accuracy of OCT for detecting TCFA was relatively higher than that of VH-IVUS (79.0 vs. 76.5%) (37). However, neither modality alone is sufficient to provide detailed information regarding the vulnerability (38). VH-IVUS cannot measure the fibrous cap thickness accurately while OCT is not able to quantify the necrotic core because of its poor penetration. The combined use of them may be a feasible approach that can markedly improve TCFA identification (37).

### Clinical Implications and Future Perspectives

The present study showed that QFR had a high negative predictive value (84.6%) in ruling out OCT-TCFA and hence may serve as an initial screening tool for high-risk plaques in stable and NSTE-ACS patients. Culprit lesions of NSTEMI are often less severe than that of STEMI and sometimes may be ambiguous to identify. In this clinical scenario, QFR might provide important information about plaque instability and infarct-related arteries before using intravascular techniques. In addition, QFR may be helpful in the assessment of non-culprit lesions in ACS patients. A recent proof-of-concept study also demonstrated the reliability and prognostic value of QFR computation in non-culprit lesions of patients with STEMI and multivessel disease (39). Similarly, the OCT sub-study of the COMPLETE trial (Complete vs. Culprit-Only Revascularization to Treat Multi-Vessel Disease After Early PCI for STEMI) also showed that obstructive non-culprit lesions are more commonly to have vulnerable characteristics than non-obstructive ones (40). Interestingly, we found a QFR value of ≤0.86 as the best cutoff for predicting OCT-TCFA, which was slightly higher than the threshold (0.80) commonly used to determine ischemia. This variation may largely result from the facts that a certain proportion of non-ischemic lesions (e.g., >25% in diabetic patients) represent OCT-TCFA (41), and our included lesions are mild-to-moderate in angiographic severity. It was also supported by a report by Hakeem et al. (42) which identified FFR cutoffs of <0.84 for predicting major adverse cardiac events in ACS patients while <0.81 in stable patients. Accordingly, in our selected population, if QFR is >0.86, high-risk plaque features are less likely to exist and further intravascular evaluation can be deferred.

Both functional severity and morphological features are indicators of plaque vulnerability and they may affect each other. In the setting of an abnormal FFR, altered fluid dynamics and shear stress increased the probability of plaque rupture, and inflammatory pathways activation (43, 44). Unstable plaques are prone to develop fissures and thrombosis on their surface, both of which may contribute to an increased loss of fluid energy and eventually affect the hemodynamic outcome. Meanwhile, physiology and anatomy seem to play an independent and irreplaceable role in predicting the occurrence of future events. Plaques with both adverse hemodynamic and morphological characteristics exhibited a significantly higher risk for subsequent ACS than those having only one (hazard ratio: 3.22) or neither of them (hazard ratio: 11.75) (45). Although non-ischemic lesions can be safely treated with medication (46), a recent study suggested that these lesions in diabetic patients may have an increased risk in future adverse events if they present OCT-TCFA (41). However, there remains controversy over how to utilize intravascular imaging and FFR in the early identification and preventive intervention of high-risk plaques (47, 48). Therefore, a comprehensive understanding of “vulnerable plaques,” and even “vulnerable patients” needs to be addressed in subsequent studies (49).

### Limitations

Some limitations should be considered when interpreting the results of the present study. First, the QFR value was retrospectively obtained although the included patients were derived from prospective studies at a single center. The QFR computation was conducted by two well-trained technicians blinded to clinical characteristics and intravascular imaging data; however, the lack of an independent core laboratory could increase the susceptibility to bias. Second, no invasive FFR measurement was performed concomitantly with the intravascular imaging evaluation and hence the concordance of QFR vs. FFR in NSTE-ACS culprit lesions and its impact on our results was unclear. Finally, the clinical value of QFR was not assessed due to our non-randomized design. Further longitudinal studies with large samples are warranted to examine whether there is a causal relationship cannot be established between QFR and plaque vulnerability.

## Conclusions

The functional severity stratified by QFR was associated with the prevalence of OCT-TCFA in angiographically mild-to-intermediate lesions from stable and NSTE-ACS patients. The QFR might be a useful tool for ruling out high-risk plaques without using any pressure wires or vasodilators. VH-IVUS-defined plaque instability, such as plaque compositions and TCFAs, may fail to influence the hemodynamic outcome of coronary lesions. Further longitudinal studies are needed to elucidate whether QFR-based decision-making might translate into improved clinical outcomes in patients with coronary artery disease.

## Data Availability Statement

The raw data supporting the conclusions of this article will be made available by the authors, without undue reservation.

## Ethics Statement

The studies involving human participants were reviewed and approved by Institutional Ethics Committee for Clinical Research of Zhongda Hospital, Affiliated to Southeast University. The patients/participants provided their written informed consent to participate in this study.

## Author Contributions

WZ and RS contributed to the data collection, analysis, and interpretation. WZ wrote the first draft of the manuscript. XZ, YQ, and ZJ contributed to data collection. YS and RZ contributed to data analysis. WZ and GM contributed to the study conception and design. All authors contributed to manuscript revision, read, and approved the final version of the manuscript.

## Conflict of Interest

The authors declare that the research was conducted in the absence of any commercial or financial relationships that could be construed as a potential conflict of interest.
