# Supplementary material for: The Association Between Quantitative Flow Ratio and Intravascular Imaging-defined Vulnerable Plaque Characteristics in Patients With Stable Angina and Non-ST-segment Elevation Acute Coronary Syndrome
Source: Front Cardiovasc Med. 2021 Jun 30;8:690262. doi: 10.3389/fcvm.2021.690262 (PMC8278311; doi:10.3389/fcvm.2021.690262)
Supplement: Supplementary file 1 [file Data_Sheet_1.pdf]

Supplementary Material

Supplementary Table 1. Baseline characteristics of VH-IVUS cohort

	VH-IVUS cohort
Clinical and demographic characteristics	
Patient No.	40
Age, years	63.8 ± 10.1
Male	23 (57.5)
Body mass index, kg/m^2	24.8 ± 4.8
Hypertension	29 (72.5)
Diabetes mellitus	10 (25.0)
Dyslipidemia	11 (27.5)
Smoking	7 (17.5)
Previous MI	1 (2.5)
Previous PCI	3 (7.5)
LVEF, %	68.3 (65.3–74.8)
Triglyceride, mmol/L	1.3 (1.0–2.1)
Total cholesterol, mmol/L	4.3 (3.4–4.9)
LDL cholesterol, mmol/L	2.4 (1.9–3.1)
HDL cholesterol, mmol/L	1.2 (1.0–1.4)
Symptoms	
Stable angina	19 (47.5)
Unstable angina	20 (50.0)
NSTEMI	1 (2.5)
Medications	
Aspirin	9 (22.5)
Statins	8 (20.0)
β–blockers	4 (10.0)
ACEI/ARB	14 (35.0)
Calcium channel blockers	13 (32.5)
Interrogated vessel characteristics	
Lesion No.	40
Lesion location	
LAD	32 (80.0)
LCX	3 (7.5)
RCA	5 (12.5)
3D–QCA	
Diameter stenosis, %	45.3 (36.8–48.5)
Lesion length, mm	19.8 (11.9–25.8)
MLD, mm	1.6 (1.5–1.9)
QFR	0.88 (0.83–0.93)

Values are expressed as n (%) or mean ± standard deviation or median (interquartile range).

ACEI, angiotensin converting enzyme inhibitor; ARB, angiotensin receptor inhibitor; HDL, high-density lipoprotein; LAD, left anterior descending artery; LCX, left circumflex artery; LDL, low-density lipoprotein; LVEF, left ventricular ejection fraction; MI, myocardial infarction; MLD, minimal lumen diameter; NSTEMI, non-ST-segment elevation myocardial infarction; PCI, percutaneous coronary intervention; QCA, quantitative coronary angiography; QFR, quantitative flow ratio; RCA, right coronary artery; VH-IVUS, virtual histology intravascular ultrasound.

Supplementary Table 2. Lesion characteristics according to the presence or absence of OCT-TCFA

	OCT-TCFA		
	Yes (n = 23)	No (n = 60)	p-value
Lesion location			0.210
LAD	16 (69.6)	46 (76.7)	
LCX	5 (21.7)	5 (8.3)	
RCA	2 (8.7)	9 (15.0)	
3D-QCA			
Diameter stenosis, %	49.1 (39.2–73.8)	38.1 (33.0–44.1)	0.001
Lesion length, mm	19.6 (12.3–28.6)	15.6 (11.0–20.8)	0.037
MLD, mm	1.3 (0.8–1.9)	1.6 (1.4–1.9)	0.038
QFR	0.83 (0.73–0.88)	0.92 (0.86–0.96)	0.002
OCT findings			
MLA, mm2	2.35 (1.64–3.13)	2.89 (2.07–3.81)	0.092
Area stenosis, %	65.9 ± 13.3	59.1 ± 12.6	0.035
Lipid-rich plaque	23 (100.0)	51 (85.0)	0.057
Maximum lipid arc, °	330 (220–360)	220 (180–310)	0.023
Lipid length, mm	20.0 (14.0–22.0)	12.0 (8.0–21.0)	0.037
Lipid index, ° × mm	2720 (1970–3895)	1901 (880–3032)	0.045
Thinnest FCT, µm	50 (30–60)	150 (100–190)	<0.001
Plaque rupture	5 (21.7)	0 (0.0)	0.001
Plaque erosion	8 (34.8)	2 (3.3)	<0.001
Thrombus	12 (52.2)	3 (5.0)	<0.001
Calcification	13 (56.5)	24 (40.0)	0.175
Calcified nodule	0 (0.0)	5 (8.3)	0.316
Microchannel	10 (43.5)	19 (31.7)	0.312
Macrophage accumulation	12 (52.2)	22 (36.7)	0.199
Cholesterol crystal	10 (43.5)	19 (31.7)	0.312

Values are expressed as n (%) or mean ± standard deviation or median (interquartile range).

FCT, fibrous cap thickness; MLA, minimal lumen area; OCT, optical coherence tomography; TCFA, thin-capped fibroatheroma; other abbreviations as Supplementary Table 1.

Supplementary Table 3. Lesion characteristics according to the presence or absence of VH-TCFA

	VH-TCFA		
	Yes (n = 6)	No (n = 34)	p-value
Lesion location			1.000
LAD	5 (83.3)	27 (79.4)	
LCX	0 (0.0)	3 (8.8)	
RCA	1 (16.7)	4 (11.8)	
3D-QCA			
Diameter stenosis, %	46.5 (35.5–48.8)	43.7 (36.8–48.6)	0.733
Lesion length, mm	13.6 (11.4–24.9)	20.2 (12.0–26.1)	0.495
MLD, mm	1.6 (1.5–1.9)	1.7 (1.5–1.9)	0.804
QFR	0.89 (0.84–0.94)	0.88 (0.82–0.94)	0.732
Grayscale IVUS findings			
EEM CSA, mm^2	13.1 (11.1–16.4)	12.3 (10.5–15.4)	0.596
Plaque + media, mm^2	9.1 (7.5–9.4)	8.6 (7.0–11.1)	0.820
Plaque burden, %	67.7 (58.4–71.5)	69.9 (64.5–77.1)	0.248
MLA, mm^2	4.1 (3.3–6.9)	3.8 (3.2–4.5)	0.343
Reference EEM CSA, mm^2	13.7 (11.0–16.6)	13.3 (11.6–15.5)	0.895
Remodeling index	1.01 (0.90–1.07)	0.89 (0.80–1.08)	0.353
Positive remodeling	2 (33.3)	11 (32.4)	1.000
Negative remodeling	2 (33.3)	20 (58.8)	0.381
VH-IVUS findings			
Fibrous tissue, mm^2	2.7 (2.1–3.6)	2.7 (1.9–4.9)	0.940
Fibrous tissue, %	57.1 (48.8–63.0)	60.2 (45.8–66.8)	0.622
Fibrofatty tissue, mm^2	1.0 (0.3–2.0)	1.4 (0.6–2.1)	0.353
Fibrofatty tissue, %	15.2 (9.0–31.5)	22.5 (15.0–32.5)	0.256
Necrotic core, mm^2	1.0 (0.6–1.3)	0.5 (0.2–1.1)	0.217
Necrotic core, %	21.0 (11.2–25.2)	9.7 (5.1–19.1)	0.140
Dense calcium, mm^2	0.2 (0.0–0.4)	0.1 (0.0–0.4)	0.664
Dense calcium, %	4.2 (0.5–7.9)	1.9 (0.0–6.1)	0.358

Values are expressed as n (%) or median (interquartile range).

CSA, cross-sectional area; EEM, external elastic membrane; other abbreviations as Supplementary Tables 1 and 2.

Supplementary Table 4. Diagnostic performance of QFR for the evaluation of plaque morphology

	Cutoff	Sensitivity, %	Specificity, %	+LR	–LR	PPV, %	NPV, %	Accuracy, %
OCT-TCFA	≤0.86	65.2 (42.7–83.6)	73.3 (60.3–83.9)	2.5 (1.5–4.1)	0.5 (0.3–0.8)	48.4 (35.9–61.1)	84.6 (75.5–90.8)	71.1 (60.1–80.5)
OCT-MLA <3.5 mm^2	≤0.94	82.5 (70.9–91.0)	65.0 (40.8–84.6)	2.4 (1.3–4.3)	0.3 (0.1–0.5)	88.1 (80.2–93.2)	54.2 (38.7–68.9)	78.3 (67.9–86.6)
Plaque burden ≥70%	≤0.92	96.0 (80.0–99.9)	45.8 (25.6–67.2)	1.8 (1.2–2.6)	0.1 (0.01–0.6)	64.9 (55.9–72.9)	91.7 (60.6–98.8)	71.4 (56.7–83.4)
IVUS-MLA <4 mm^2	≤0.86	58.6 (38.9–76.5)	80.0 (56.3–94.3)	2.9 (1.2–7.4)	0.5 (0.3–0.8)	81.0 (62.7–91.5)	57.1 (45.1–68.4)	67.4 (52.5–80.1)

Values are expressed as absolute numbers (95% CI).

CI, confidence interval; +LR, positive likelihood ratio; –LR, negative likelihood ratio; NPV, negative predictive value; PPV, positive predictive value; other abbreviations as Supplementary Tables 1 and 2.

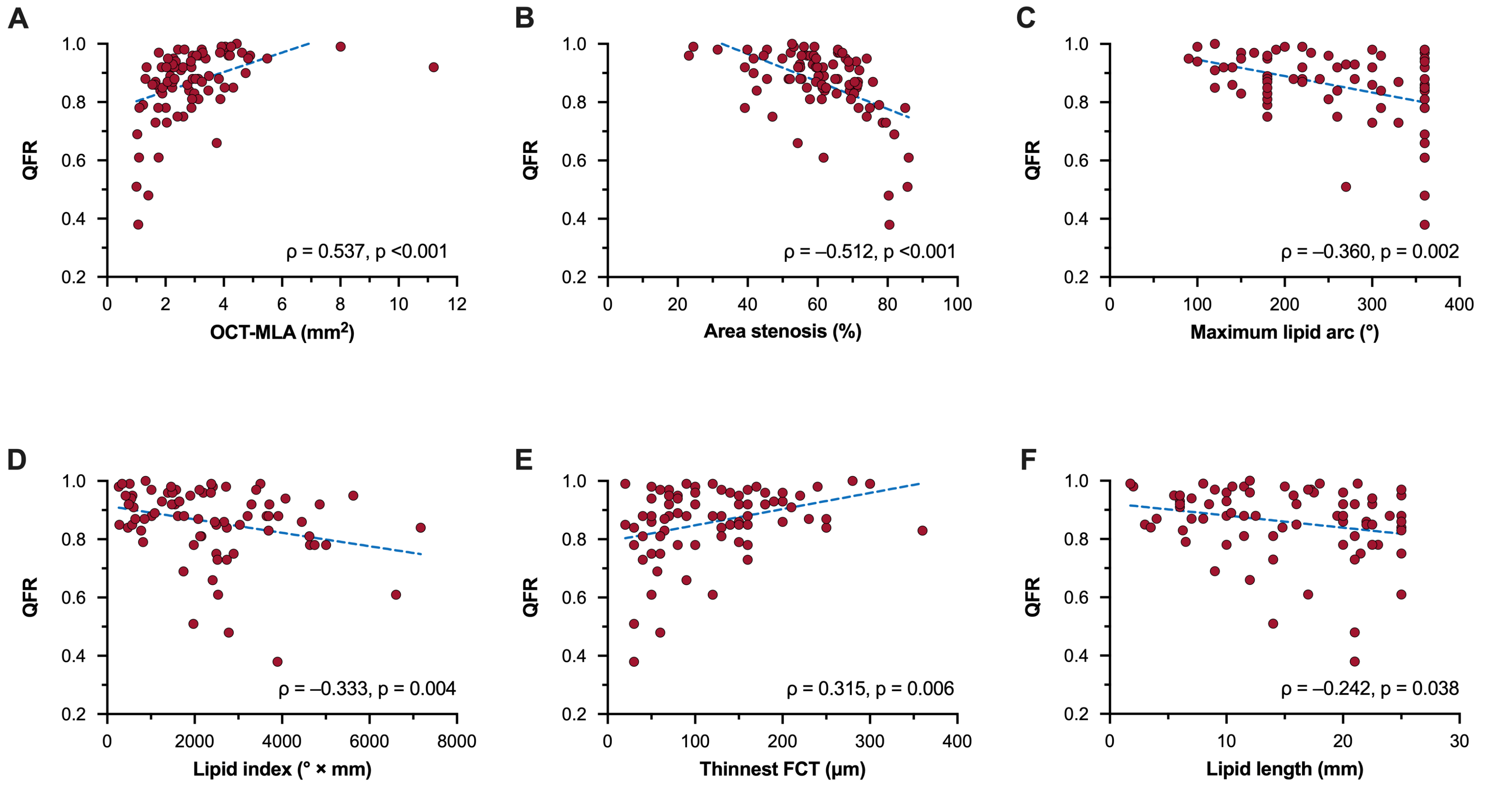

Supplementary Figure 1. Relationship between QFR and OCT-derived parameters. The Spearman's correlation analysis indicated that QFR was strongly related to OCT-MLA (A) and % area stenosis (B); QFR was modestly related to maximum lipid arc (C), lipid index (D), and thinnest FCT (E); QFR was weakly related to lipid length (F). Abbreviations as Supplementary Tables 1 and 2.

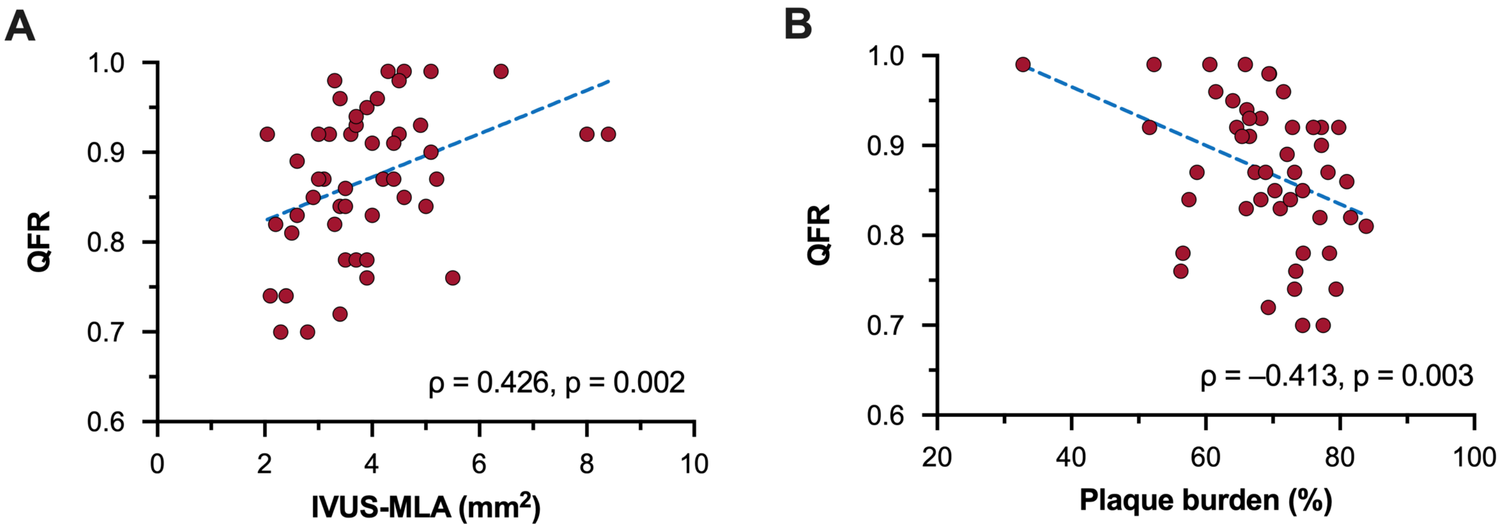

Supplementary Figure 2. Relationship between QFR and IVUS-derived parameters. QFR was modestly related to IVUS-MLA (A) and plaque burden (B). Abbreviations as Supplementary Tables 1 and 2.
